# Magnetic resonance imaging of white matter in Alzheimer's disease: a global bibliometric analysis from 1990 to 2022

**DOI:** 10.3389/fnins.2023.1163809

**Published:** 2023-05-25

**Authors:** Jin-huan Yue, Qin-hong Zhang, Xu Yang, Peng Wang, Xu-Chen Sun, Shi-Yan Yan, Ang Li, Wei-Wei Zhao, Dan-Na Cao, Yang Wang, Ze-Yi Wei, Xiao-Ling Li, Lu-Wen Zhu, Guanhu Yang, Jeffrey Zhongxue Mah

**Affiliations:** ^1^Department of Tuina, Acupuncture and Moxibustion, Shenzhen Jiuwei Chinese Medicine Clinic, Shenzhen, China; ^2^Graduate School of Heilongjiang University of Chinese Medicine, Harbin, China; ^3^Department of Oncology, First Affiliated Hospital of Heilongjiang University of Chinese Medicine, Harbin, China; ^4^International Acupuncture and Moxibustion Innovation Institute, School of Acupuncture-Moxibustion and Tuina, Beijing University of Chinese Medicine, Beijing, China; ^5^Sanofifi-Aventis China Investment Co., Ltd, Beijing, China; ^6^MSD R&D (China) Co., Ltd, Beijing, China; ^7^Division of CT and MRI, First Affiliated Hospital of Heilongjiang University of Chinese Medicine, Harbin, China; ^8^Department of Rehabilitation, Second Affiliated Hospital of Heilongjiang University of Chinese Medicine, Harbin, China; ^9^Department of Specialty Medicine, Ohio University, Athens, OH, United States; ^10^Consotherapy Oncology Center, Vitality University, Hayward, CA, United States

**Keywords:** Alzheimer's disease, white matter, magnetic resonance imaging, CiteSpace, VOSviewer, bibliometric analysis

## Abstract

**Background:**

Alzheimer's disease (AD) is a common, progressive, irreversible, and fatal neurodegenerative disorder with rapidly increasing worldwide incidence. Although much research on magnetic resonance imaging (MRI) of the white matter (WM) in AD has been published, no bibliometric analysis study has investigated this issue. Thus, this study aimed to provide an overview of the current status, hotspots, and trends in MRI of WM in AD.

**Methods:**

We searched for records related to MRI studies of WM in AD from 1990 to 2022 in the Web of Science Core Collection (WOSCC) database. CiteSpace (version 5.1.R8) and VOSviewer (version 1.6.19) software were used for bibliometric analyses.

**Results:**

A total of 2,199 articles were obtained from this study. From 1990 to 2022, the number of published articles showed exponential growth of y = 4.1374e^0.1294x^, with an average of 17.9 articles per year. The top country and institutions were the United States and the University of California Davis, accounting for 44.52 and 5.32% of the total studies, respectively. The most productive journal was Neurology, and the most co-cited journal was Lancet Neurology. Decarli C was the most productive author. The current research frontier trend focuses on the association between small vessel disease and AD, the clinical application and exploration of diffusion MRI, and related markers.

**Conclusion:**

This study provides an in-depth overview of publications on MRI of WM in AD, identifying the current research status, hotspots, and frontier trends in the field.

## 1. Introduction

Alzheimer's disease (AD) is a neurological degenerative disease with an insidious and progressive onset (Ben Miled et al., 2020) and is considered a serious problem for both individual health and government healthcare systems worldwide (Nabizadeh et al., 2022). White matter (WM) lesions are a common finding in AD and may contribute to dementia severity. Although previous studies have explored the neural mechanisms underlying AD, these mechanisms are not well understood.

In recent years, with the development of neuroimaging technologies, magnetic resonance imaging (MRI) has provided a new perspective to enhance our understanding of AD mechanisms. It provides valuable insights into the structure and function of neural networks (Chard et al., 2021). Many studies have used MRI to investigate WM changes and pathological features of AD, including WM lesions (Vermeer et al., 2003), mild cognitive impairment (Annweiler et al., 2013), Pittsburgh compound B (PiB)-induced WM pathology (Glodzik et al., 2015), diffuse MRI (Tseng et al., 2022), small vessel disease (Acharya et al., 2019), and risk factors (Artero et al., 2004). Although these studies have enhanced our understanding of the imaging mechanism of AD using MRI, little attention has been paid to the current research status, hotspots, and frontier trends in this field.

In recent years, bibliometric analysis has been widely used to explore academic publications (Kim and Park, 2021). It applies mathematical and statistical methods, bibliometrics, and data-mining algorithms to visualize the co-citation network of scientific research and identify trends and structures within a knowledge domain (Kim and Park, 2021). By interpreting the information in the network map, researchers can quickly and accurately understand the research status, hotspots, and trends of this topic in the field (Chen, 2017). In this study, we conducted a bibliometric analysis of publications related to MRI of WM in patients with AD in the Web of Science Core Collection (WOSCC) between 1990 and 2022. This study provides insights and perspectives into the literature on the MRI of WM in AD to better understand the current research status, hotspots, and frontier trends.

## 2. Methods

### 2.1. Study design

This study was retrospectively reviewed using bibliometric analysis. We performed a literature search using the WOSCC database. Bibliometric analysis was performed using VOSviewer and CiteSpace software. The number of publications, countries, institutions, authors, keywords, and references, as well as their associations, were analyzed.

### 2.2. Data acquisition

The search strategy was as follows: TS = (“Alzheimer's^*^ disease” AND “white matter” AND “MRI” AND “brain”). The time span was between 1990 and 2022. Only the original articles and reviews published in English were included. The full records and all references are explained in a plain text format. Consequently, 2199 articles were obtained.

### 2.3. Statistical analysis

All available data were collected in WOSCC and imported to Microsoft Excel 2022, CiteSpace (version 5.1.R8) (Chen, 2017), and VOSviewer (version 1.6.19) (Van Eck and Waltman, 2010) for performing bibliometric analysis.

Microsoft Excel 2022 was used to draw a trend chart of the annual output of the 2199 records. We used VOSviewer to construct a network of countries, institutions, authors, co-cited journals, and keyword co-ocurrence. We applied CiteSpace to perform keyword clustering and keyword citation burst. The node in each map indicates a country, institution, author, co-cited journal, or a keyword. The size of the node (country, institution, or author) was proportional to the number of publications. The larger the node, the greater is the number of publications. The links between the nodes indicate their collaboration. For co-cited journals, the size of the node is proportional to the number of total citations; the larger the node, the greater is the number of total citations. The line between them refers to the co-citation of the journals. The size of the keywords is proportional to their frequency, with a larger node signifying a higher keyword frequency. The connection between them indicates keyword co-occurrence.

We set the parameters of CiteSpace as follows: time span (1990–2022), time slice (3 years), node type (country, institution, author, keyword, cited reference, or cited journal), node threshold (topn = 50), and pruning (pathfinder or MST algorithm). Detailed information is available at http://cluster.cis.drexel.edu/~cchen/citespace/ and https://www.vosviewer.com/.

## 3. Results

### 3.1. The trends of publication outputs

**Figure 1:**
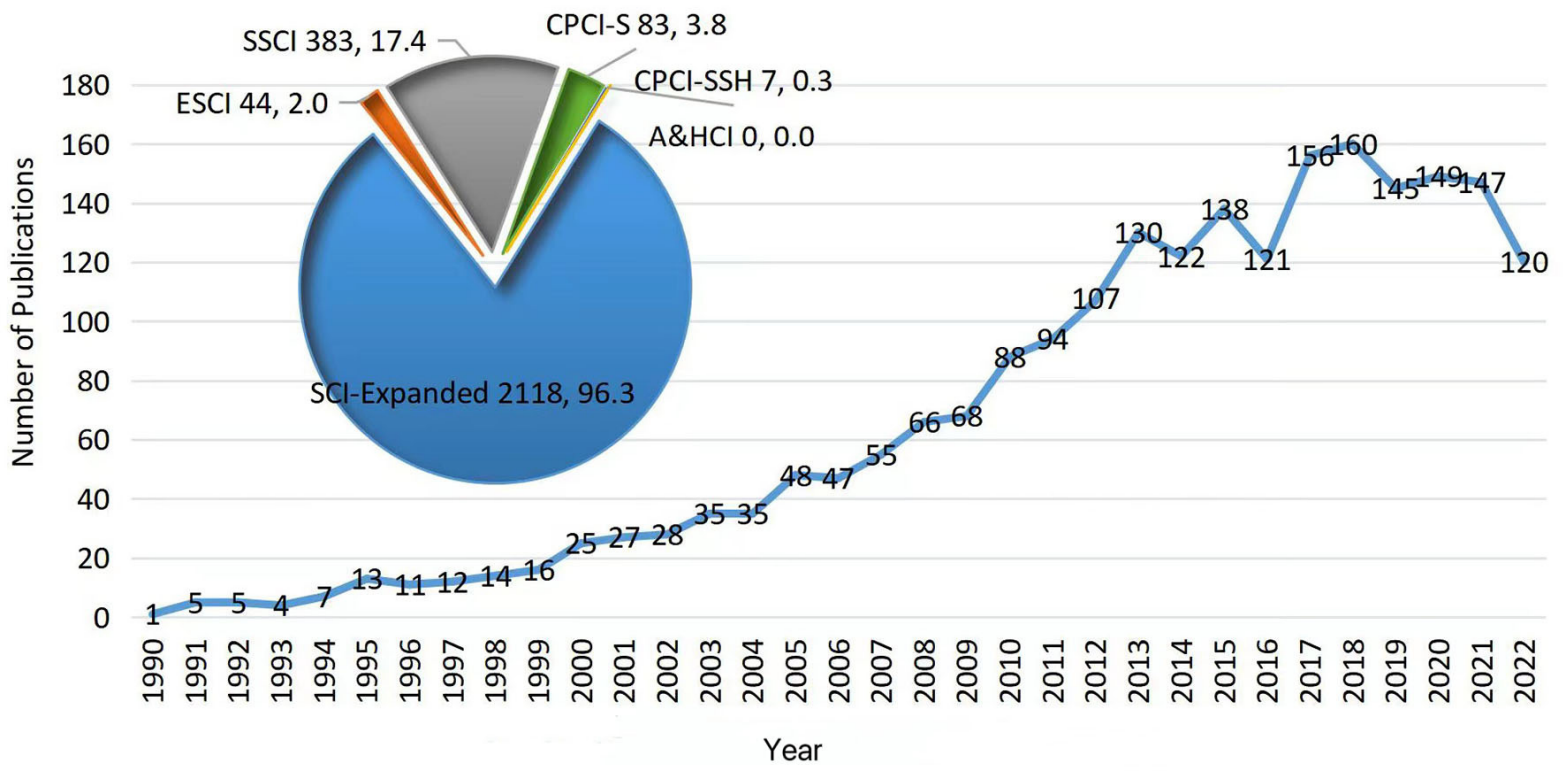
The number and trend of annual publication.

The annual number of publications is shown in Figure 1. As can be seen from the figure, the number of publications in the field of MRI studies on WM in AD has shown an increasing trend, and the fitted curve index was y = 4.1374e^0.1294x^. The first such study was published in 1990. The overall number of publications fluctuates from 1990 to 2018, peaking in 2018. In particular, the number of publications fluctuated slowly and steadily from 2018 to 2022. However, all of these publications were over 145 annually, except for 2022, because the search date was 24 October 2022, and the annual publications in 2022 were incomplete.

### 3.2. Distribution and collaboration of countries

**Figure 2:**
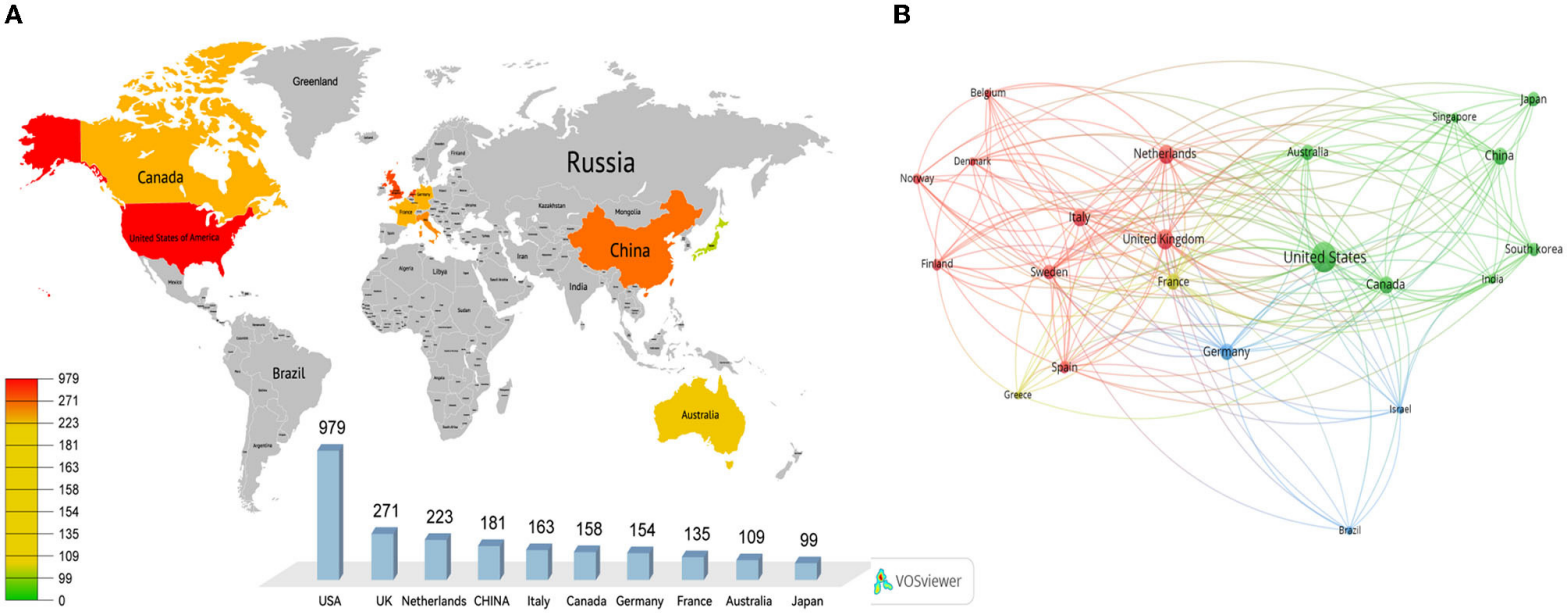
Cooperative network of country distribution **(A)** and collaboration **(B)**.

The data showed that 26 countries contributed to research on MRI studies of WM in AD. Figure 2A shows a world map of productive countries. The top 10 most productive countries are listed in Table 1. The United States was the most productive country (*n* = 979 articles), accounting for approximately 44.52% of the total literature, followed by the United Kingdom (271 articles), Netherlands (223 articles), China (181 articles), Italy (163 articles), Canada (158 articles), Germany (154 articles), France (135 articles), Australia (109 articles), and Japan (99 articles). The country with the highest centrality was the United States (0.25), followed by the United Kingdom (0.19), Canada (0.18), and France (0.10). This shows that the four countries in the field of international recognition research results are higher, and their impact is greater.

**Table 1:** Top 10 productive countries and institutions.

**Ranking**	**Country**	**Frequency (articles)**	**Centrality**	**Ranking**	**Institution**	**Frequency (articles)**	**Centrality**
1	United States	979	0.25	1	Univ Calif Davis	117	0.11
2	United Kingdom	271	0.19	2	Univ Calif San Francisco	91	0.09
3	Netherlands	223	0.06	3	Univ Calif Los Angeles	77	0.11
4	China	181	0.03	4	Boston Univ	66	0.05
5	Italy	163	0.09	5	Univ College London	65	0.09
6	Canada	158	0.18	6	National Institute Accountants	57	0.02
7	Germany	154	0.05	7	Mayo Clin	56	0.09
8	France	135	0.10	8	Johns Hopkins Univ	54	0.06
9	Australia	109	0.04	9	Harvard Med Sch	52	0.01
10	Japan	99	0.01	10	Univ Toronto	51	0.05

A cooperative network for collaborations by VOSviewer is shown in Figure 2B. A total of 263 collaborations were identified among these 26 countries. The nodes of the United States, the United Kingdom, Netherlands, and China are larger, which means that they had more collaborations than other countries. This shows that many countries have focused on MRI studies of WM in AD and have formed close international collaborative networks.

### 3.3. Collaboration of institutions

**Figure 3:**
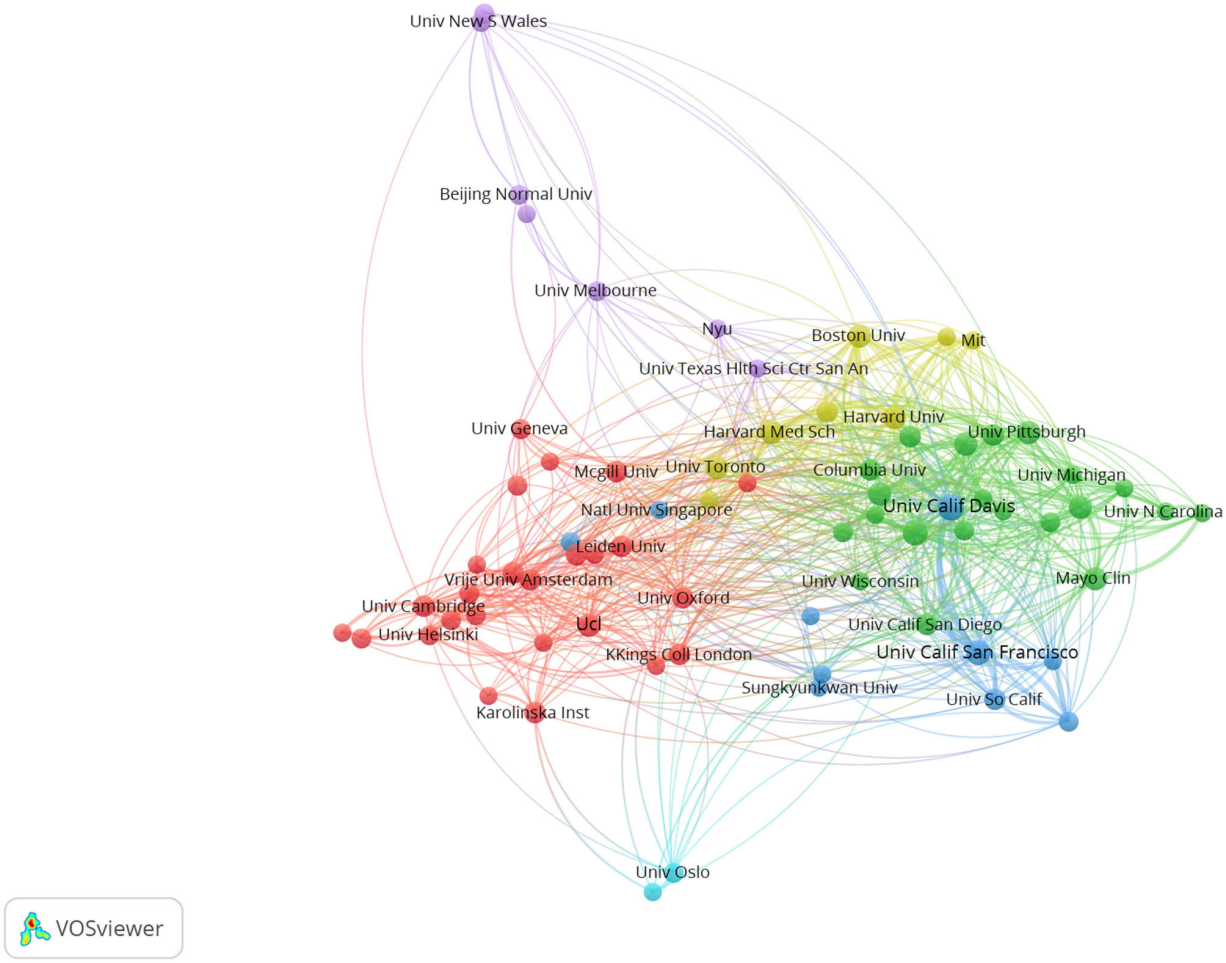
Cooperative network of institutions.

The cooperative network of institutions and their collaborations by VOSviewer are shown in Figure 3. There were 75 institutions with 725 collaborations. The top 10 countries with the highest outputs are listed in Table 1. The most productive institution was the University of California Davis (117 articles), followed by the University of California San Francisco (91 articles), and the University of California Los Angeles (77 articles). The University of California Davis and University of California Los Angeles had the highest centrality of 0.11, followed by the Mayo Clinic, University of California San Francisco, and University College London at 0.09.

### 3.4. Authors and co-cited authors

**Figure 4:**
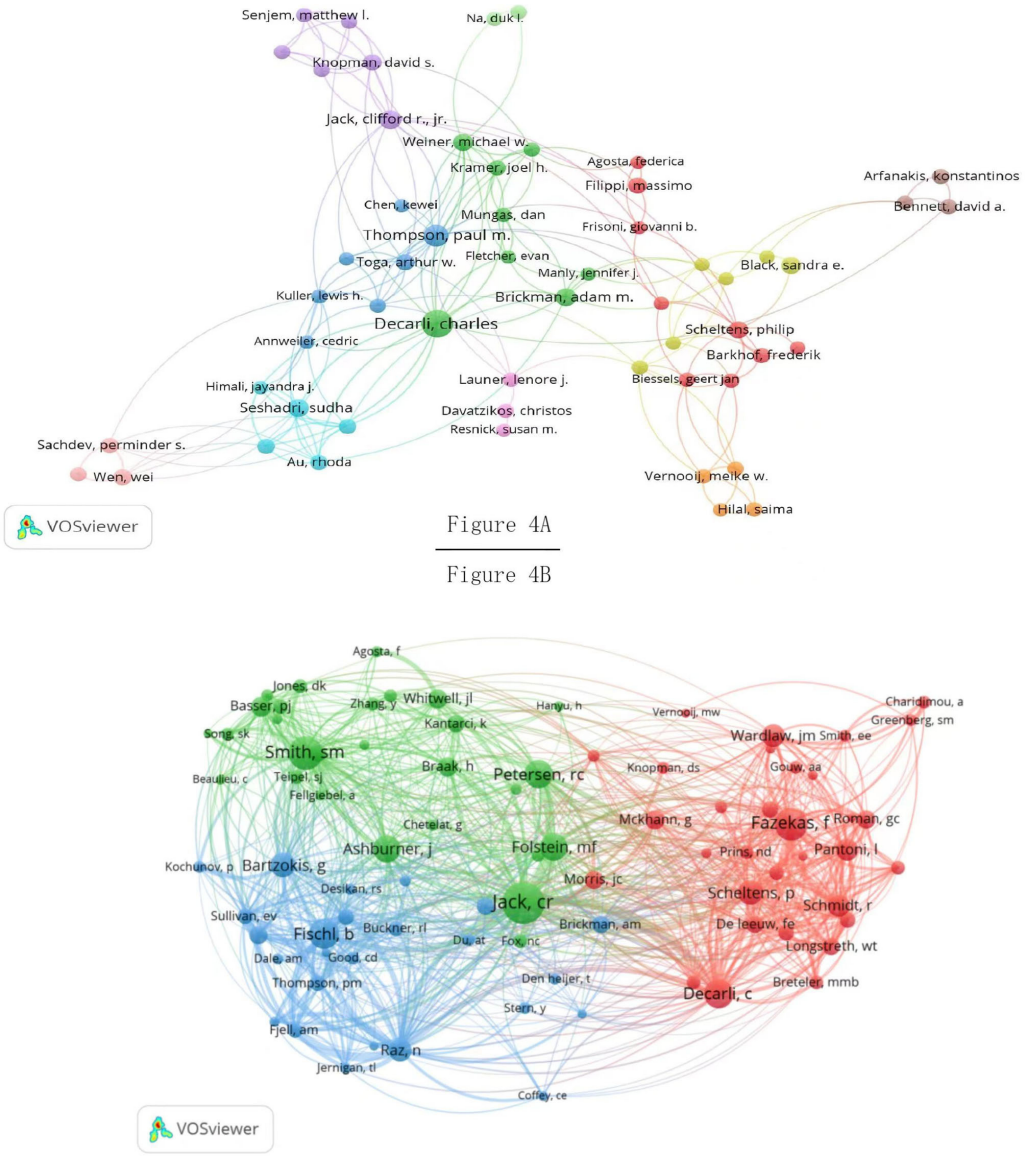
Cooperative network of productive **(A)** and co-cited **(B)** authors.

The authors' cooperative network using VOSviewer is shown in Figure 4A. A total of 61 authors and 201 collaborations between them contributed to this study. The top 10 productive authors are listed in Table 2. Decarli C had the highest output in the field with 86 publications. This was followed by Thompson PM (46 articles), Jack CR (45 articles), Scheltens P (44 articles), Weiner MW (36 articles), Barkhof F (31 articles), Toga AW (30 articles), Brickman AM (26 articles), Knopman DS (25 articles), and Seshadri S (25 articles). Scheltens P (0.34) had the highest centrality, followed by Thompson PM (0.22), Jack CR (0.14), and Weiner MW (0.10). Cooperation mainly revolves around high-yield authors and has formed good collaborative association.

**Table 2:** Top ten productive and co-cited authors.

**Productive author ranking**	**Author**	**Frequency (articles)**	**Centrality**	**Co-cited author ranking**	**Author**	**Frequency (citations)**
1	DeCarli C	86	0.09	1	Jack CR	786
2	Thompson PM	46	0.22	2	Smith SM	593
3	Jack CR	45	0.14	3	Fazekas F	557
4	Scheltens P	44	0.34	4	Fischl B	506
5	Weiner MW	36	0.10	5	Decarli C	483
6	Barkhof F	31	0.04	6	Petersen RC	467
7	Toga AW	30	0.09	7	Folstein MF	443
8	Brickman AM	26	0.08	8	Ashburner J	429
9	Knopman DS	25	0.01	9	Bartzokis G	388
10	Seshadri S	25	0.02	10	Scheltens P	378

The results of the authors' co-citation analysis network by using VOSviewer are shown in Figure 4B. Among the 34,280 authors, 86 authors (0.25%) were cited more than 100 times, 31 contributors (0.09%) were cited at least 300 times, and 13 authors (0.04%) were cited more than 500 times. The top ten co-cited authors were cited more than 350 times (Table 2), among which Jack CR was the most cited author (786 citations), followed by Smith SM (593 citations), Fazekas F (557 citations), Fischl B (506 citations), Decarli C (483 citations), Petersen RC (467 citations), Folstein MF (443 citations), Ashburner J (429 citations), Bartzokis G (388 citations), and Scheltens P (378 citations).

### 3.5. Journals and co-cited journals

**Figure 5:**
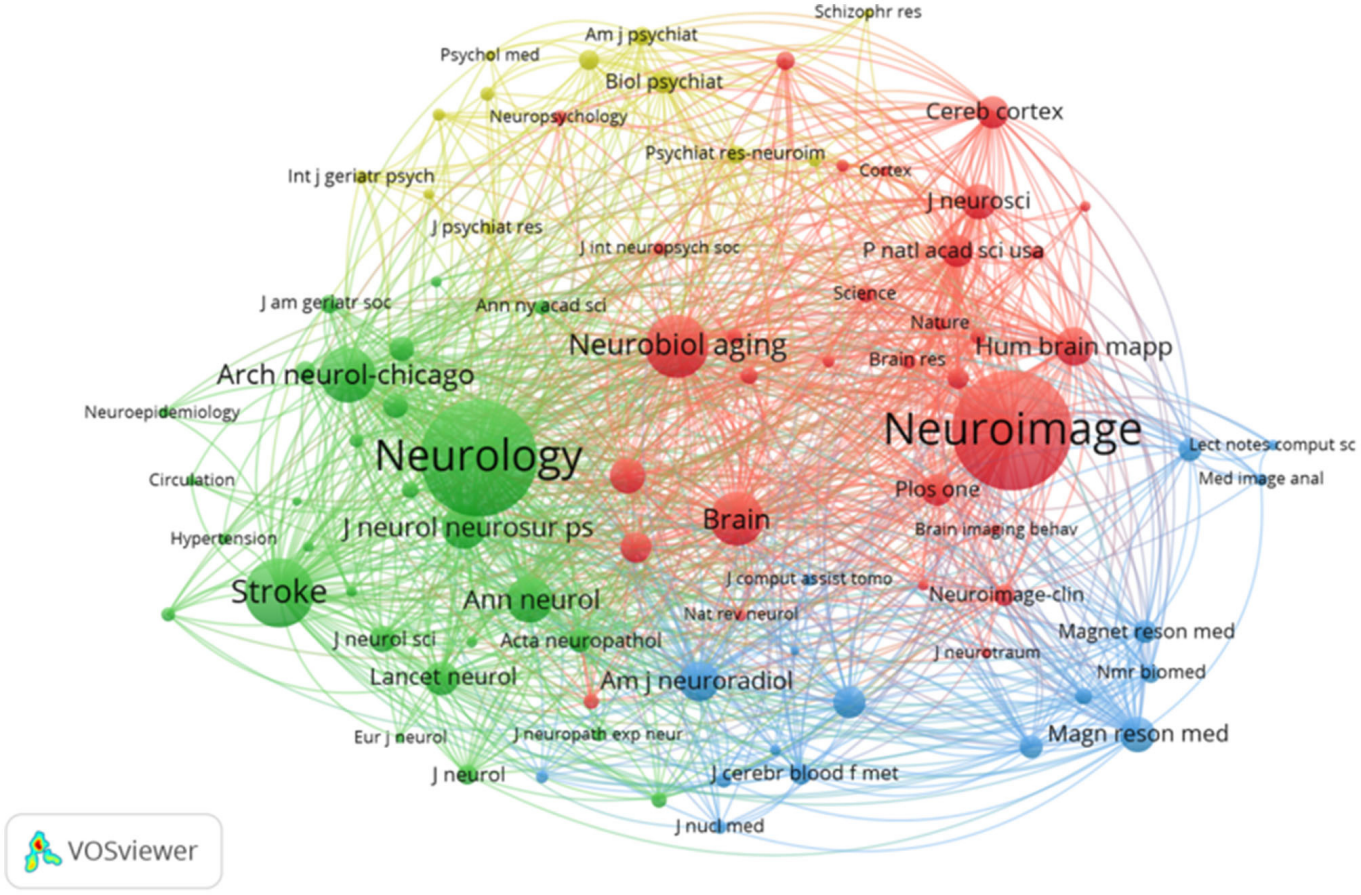
Cooperative network of co-cited journals.

The cooperative network of co-cited journals by VOSviewer is shown in Figure 5. The top 10 journals with the highest outputs are listed in Table 3. Neurology (162 articles) was the most productive journal, followed by Neuroimage (129 articles), Neurobiology of Aging (110 articles), and Journal of Alzheimer's Disease (89 articles). Neurology (impact factor (IF), 11.800) and stroke (IF, 10.170) were the most influential journals.

**Table 3:** Top ten productive and co-cited journals.

**Ranking**	**Journal**	**Frequency** **(articles)**	**IF(2021)**	**Q(2021)**	**Ranking**	**Co-cited journal**	**Frequency (citations)**	**Centrality**	**IF(2021)**	**Q(2021)**
1	Neurology	162	11.800	Q1	1	Neurology	1079	0.12	11.800	Q1
2	NeuroImage	129	7.400	Q1	2	NeuroImage	863	0.09	7.400	Q1
3	Neurobiology of Aging	110	5.133	Q2	3	Neurobiology of Aging	753	0.11	5.133	Q2
4	Journal of Alzheimer's Disease	89	4.160	Q2	4	Brain	740	0.06	15.255	Q1
5	Frontiers in Aging Neuroscience	65	5.702	Q1	5	Ann Neurol	671	0.07	17.274	Q1
6	PloS One	55	3.752	Q2	6	Archives of neurology	628	0.05	0.000	N/A
7	NeuroImage-Clinical	53	4.891	Q2	7	Stroke	628	0.03	10.170	Q1
8	Stroke	53	10.170	Q1	8	Journal of Neurology Neurosurgery and Psychiatry	597	0.03	13.651	Q1
9	Human Brain Mapping	48	5.399	Q1	9	PloS One	578	0.02	3.752	Q2
10	Journal of the Neurological Sciences	38	4.553	Q2	10	Journal of Alzheimer's Disease	564	0.01	4.160	Q2

IF, impact factor; Q, quartile; Q1, first quartile; Q2, second quartile.

The top 10 journals with the most frequent co-citations are listed in Table 3. The most frequently co-cited journal was Neurology (1,079 citations, IF, 11.800), followed by Neuroimage (863 citations, IF, 7.400), Neurobiology of Aging (753 citations, IF, 5.133), and Brain (740 citations, IF, 15.255). The journals with high centrality were Neurology (0.12) and the Neurobiology of Aging (0.11). In addition, Lancet Neurology had the highest impact (IF, 59.935). Journals with high outputs and frequent citations, especially for Neurology and Neuroimage, play an important role in this field.

### 3.6. Research hotspots

Keywords are the high induction and central ideas of an article (Small, 1973; Chen, 2010). The research hotspots in MRI research in the WM of AD have mainly been investigated using the keywords co-occurrence, clustering, and co-citation.

#### 3.6.1. Keyword co-occurrence and clustering

**Figure 6:**
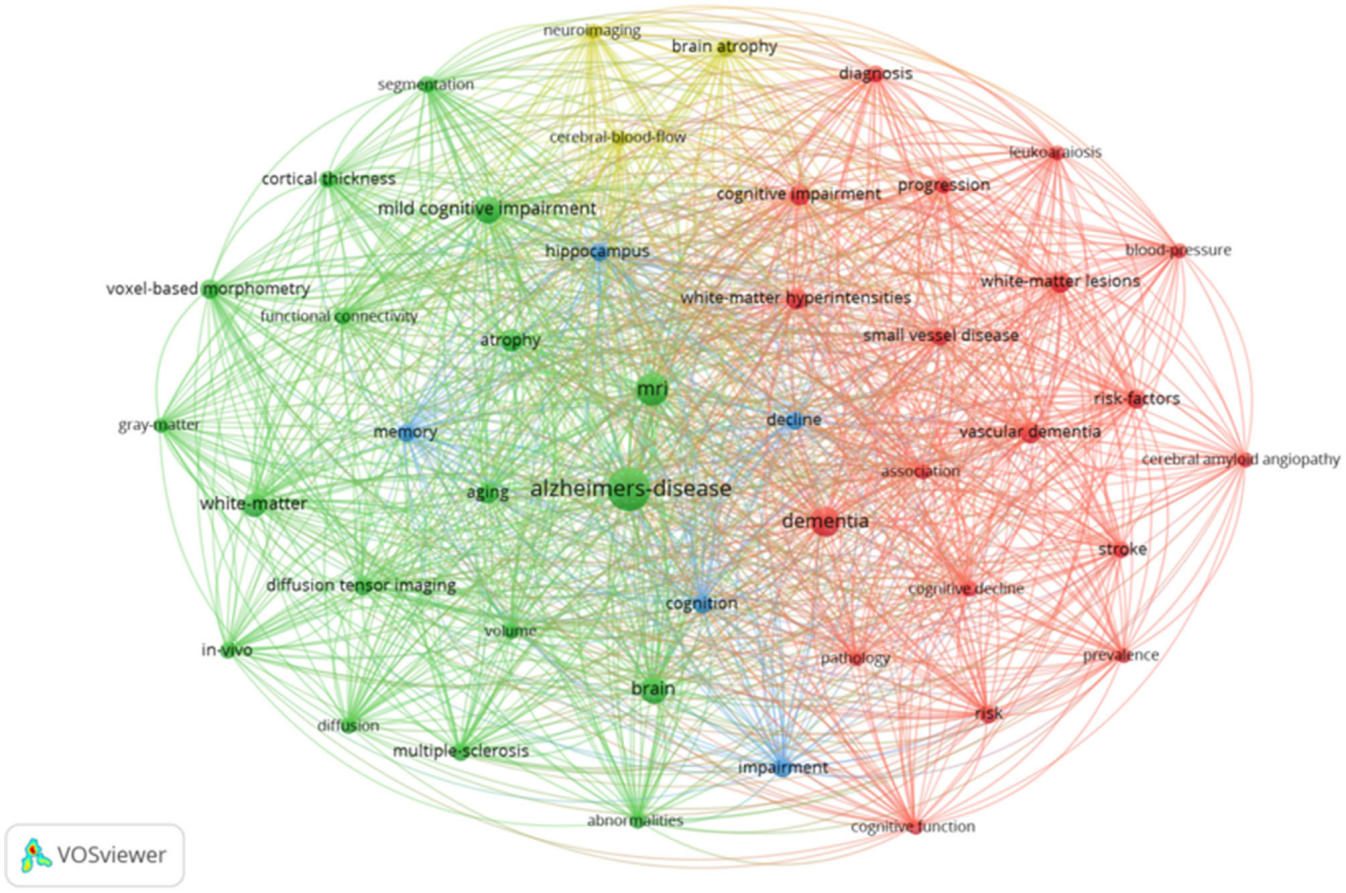
Keyword co-occurrence network.

A keyword co-occurrence network with 89 keywords and 3,403 keyword co-occurrences was built using VOSviewer (Figure 6). The top 10 keywords for frequency and centrality are listed in Table 4. AD had the highest frequency (2,031 times) with the largest corresponding node, followed by MRI (958 times), dementia (626 times), brain (582 times), WM (444 times), mild cognitive impairment (375 times), WM hyperintensity (293 times), atrophy (240 times), WM lesions (221 times), and risk factors (206 times). In terms of centrality, age (0.48) was the highest at 0.48, followed by MRI (0.44), lesions (0.35), healthy elderly subjects (0.34), and risk factors (0.31), all of which were over 0.30.

**Table 4:** Top 10 keywords with centrality and frequency.

**Ranking**	**Frequency**	**Keyword**	**Centrality**	**Ranking**	**Centrality**	**Keyword**	**Frequency**
1	2031	Alzheimer's disease	0.30	1	0.48	Age	129
2	958	MRI	0.44	2	0.44	Mri	958
3	626	Dementia	0.22	3	0.35	Lesion	176
4	448	Brain	0.18	4	0.34	Healthy elderly subject	4
5	444	White matter	0.21	5	0.31	Risk factor	206
6	375	Mild cognitive impairment	0.01	6	0.30	Alzheimer's disease	2031
7	293	White matter hyperintensity	0.06	7	0.27	Memory	133
8	240	Atrophy	0.24	8	0.25	Hyperintensity	60
9	221	White matter lesion	0.02	9	0.25	Leukoencephalopathy	9
10	206	Risk factor	0.31	10	0.24	Atrophy	240

CiteSpace software was used to build keyword clustering with Q = 0.7582 and S = 0.8097, indicating that the clustering result was scientific, reasonable, and significant. There are nine clusters of keywords as follows: #0 aging, #1 senile dementia, #2 magnetic resonance imaging, #3 mild cognitive impairment, #4 voxel-based morphometry, #5 leukoencephalopathy, #6 progression, #7 registration, and #8 cortex. Cluster numbering and font size are mainly based on the cluster size; that is, the smaller the label, the larger the cluster font, indicating that the cluster contains more keywords.

Keyword clusters are presented in Table 5. The results show that each cluster index is within a reasonable range, indicating that a single cluster is better and that the module is more homogenous. Cluster #0 contained 17 keywords with the first five feature words of aging, visual retention test, sex difference, longitudinal CT, and temporal lobe. Cluster #1 included 17 keywords with the first five feature words of senile dementia, risk, depression, atrophy, and Lewy body. Cluster #2 included 15 keywords and the first five features were MRI, leukoaraiosis, high signal lesions, DTI, and cerebral atrophy. Cluster #3 consisted of 15 keywords, and the first five ones were mild cognitive impairment, white matter hyperintensity, gait velocity, medial temporal atrophy, and older adults. Cluster #4 had 15 keywords, and the first five words were voxel-based morphometry, PET, connectivity, diffusion tensor imaging, and val(158)met genotype. Cluster #5 had 14 keywords, and the first five words were leukoencephalopathy, diffusion tensor imaging, leukoencephalopathies, epsilon 4 allele, and subcortical lesion. Cluster #6 involved 13 keywords, with the first five feature words being progression, risk factor, blood pressure, small vessel disease, and insulin resistance. Cluster #7 had 11 keywords, and the first five feature words extracted were registration, onset, ferritin, substantia nigra, and signal hyperintensity. Cluster #8 contained 11 keywords, with the first five words being the cortex, the white matter, neuroinflammation, Williams syndrome, and fractal complexity.

**Table 5:** Keywords cluster.

**ID Ranking (#)**	**Size**	**Silhouette**	**Top-term (LLR)**
0	17	0.708	aging (14.34, 0.001); visual retention test (11.29, 0.001); sex difference (11.29, 0.001); longitudinal ct (11.29, 0.001); temporal lobe (10.46, 0.005)
1	17	0.821	senile dementia (9.31, 0.005); risk (8.77, 0.005); depression (7.64, 0.01); atrophy (7.36, 0.01); lewy body (6.93, 0.01)
2	15	0.717	magnetic resonance imaging (16.41, 1.0E-4); leukoaraiosis (10.89, 0.001); high signal lesions (10.44, 0.005); dti (8.07, 0.005); cerebral atrophy (7.81, 0.01)
3	15	0.95	mild cognitive impairment (15.84, 1.0E-4); white matter hyperintensity (11.53, 0.001); gait velocity (10.02, 0.005); medial temporal atrophy (10.02, 0.005); older adults (10.02, 0.005)
4	15	0.859	voxel based morphometry (17.49, 1.0E-4); pet (12.67, 0.001); connectivity (9.83, 0.005); diffusion tensor imaging (8.86, 0.005); val(158)met genotype (6.59, 0.05)
5	14	0.858	leukoencephalopathy (28.97, 1.0E-4); diffusion tensor imaging (18.02, 1.0E-4); leukoencephalopathies (17.47, 1.0E-4); epsilon 4 allele (17.47, 1.0E-4); subcortical lesion (17.47, 1.0E-4)
6	13	0.954	progression (17.96, 1.0E-4); risk factor (15.99, 1.0E-4); blood pressure (15.02, 0.001); small vessel disease (13.79, 0.001); insulin resistance (9.27, 0.005)
7	11	0.859	registration (20.31, 1.0E-4); onset (13.53, 0.001); ferritin (13.53, 0.001); substantia nigra (13.53, 0.001); signal hyperintensity (13.53, 0.001)
8	11	0.952	cortex (21.16, 1.0E-4); white matter (16.39, 1.0E-4); neuroinflammation (11.51, 0.001); williams syndrome (10.57, 0.005); fractal complexity (10.57, 0.005)

**Figure 7:**
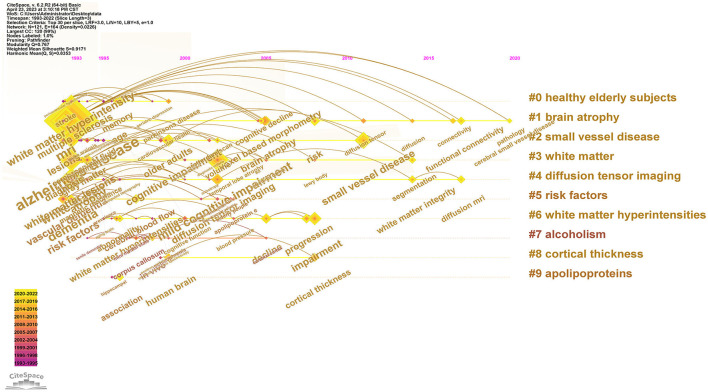
Timeline viewer of keywords cluster.

The timeline viewer of MRI in the WM of AD is drawn based on CiteSpace software (Figure 7), which visually presents the phased hotspots of this issue from the time dimension. From 1990 to 1999, research focused on the health of the elderly, and the main keywords were multiple sclerosis, geriatric depression, and Alzheimer's disease. From 1999 to 2009, the study focused on carrier protein and risk factors, and the main keywords were related to the brain, cognitive impairment, Alzheimer's disease, temporal lobe, and alcoholism. From 2009 to 2022, the research mainly focused on brain atrophy, diffusion tensor imaging, and the white matter, and the main keywords were associated with small vessel disease, functional connectivity, white matter integrity, and pathology.

#### 3.6.2. Co-cited reference

The five most frequently co-cited references are listed in Table 6, and the top five centrality references are listed in Table 7. “Neuroimaging standards for research into small vessel disease and its contribution to aging and neurodegeneration” was the most co-cited article on WM of AD by MRI with 125 citations (Wardlaw et al., 2013). It was published by Wardlaw JM in Lancet Neurology in 2013 (Wardlaw et al., 2013). This study reported neuroimaging criteria for the study of small vessel diseases and their effects on aging and neurodegeneration (Wardlaw et al., 2013), and the results have important implications for the standardization of image interpretation of common neurodegenerative pathophysiology and clinical features (Wardlaw et al., 2013). “Prevalence of cerebral white matter lesions in elderly people: A population-based magnetic resonance imaging study The Rotterdam Scan Study” was the article with the highest centrality of 0.84 (Leeuw et al., 2001). It was published by De Leeuw Fe in the Journal of Neurology Neurosurgery and Psychiatry in 2001 (Leeuw et al., 2001). The prevalence and degree of cerebral WM lesions increase with age, and women tend to have a higher degree of WM lesions than men (Leeuw et al., 2001).

**Table 6:** Top five frequently co-cited references.

**Ranking**	**Title**	**Author**	**Journal**	**Frequency (citations)**	**Year**
1	Neuroimaging standards for research into small vessel disease and its contribution to aging and neurodegeneration	Wardlaw JM	Lancet Neurology	125	2013
2	White matter hyperintensities, cognitive impairment and dementia: an update	Prins ND	Nature Reviews Neurology	64	2015
3	FSL	Jenkinson M	NeuroImage	46	2012
4	What are White Matter Hyperintensities Made of? Relevance to Vascular Cognitive Impairment	Wardlaw JM	Journal of the American Heart Association	44	2015
5	White matter hyperintensities are a core feature of Alzheimer's disease: Evidence from the dominantly inherited Alzheimer network	Lee S	Annals of Neurology	41	2016

**Table 7:** Top five co-cited references of high centrality.

**Ranking**	**Title**	**Author**	**Journal**	**Frequency**	**Year**
1	Prevalence of cerebral white matter lesions in elderly people: a population based magnetic resonance imaging study. The Rotterdam Scan Study	De Leeuw FE	Journal of Neurology Neurosurgery and Psychiatry	0.84	2001
2	Clinical correlates of white matter findings on cranial magnetic resonance imaging of 3301 elderly people - The cardiovascular health study	Longstreth WT	Stroke	0.80	1996
3	About “axial” and “radial” diffusivities	Wheeler-Kingshott CAM	Magnetic Resonance in Medicine	0.74	2009
4	White matter changes in mild cognitive impairment and AD: A diffusion tensor imaging study	Medina D	Neurobiology of Aging	0.61	2006
5	Relation between age-related decline in intelligence and cerebral white-matter hyperintensities in healthy octogenarians: a longitudinal study	Garde E	Lancet	0.35	2000

### 3.7. Research trend

**Figure 8:**
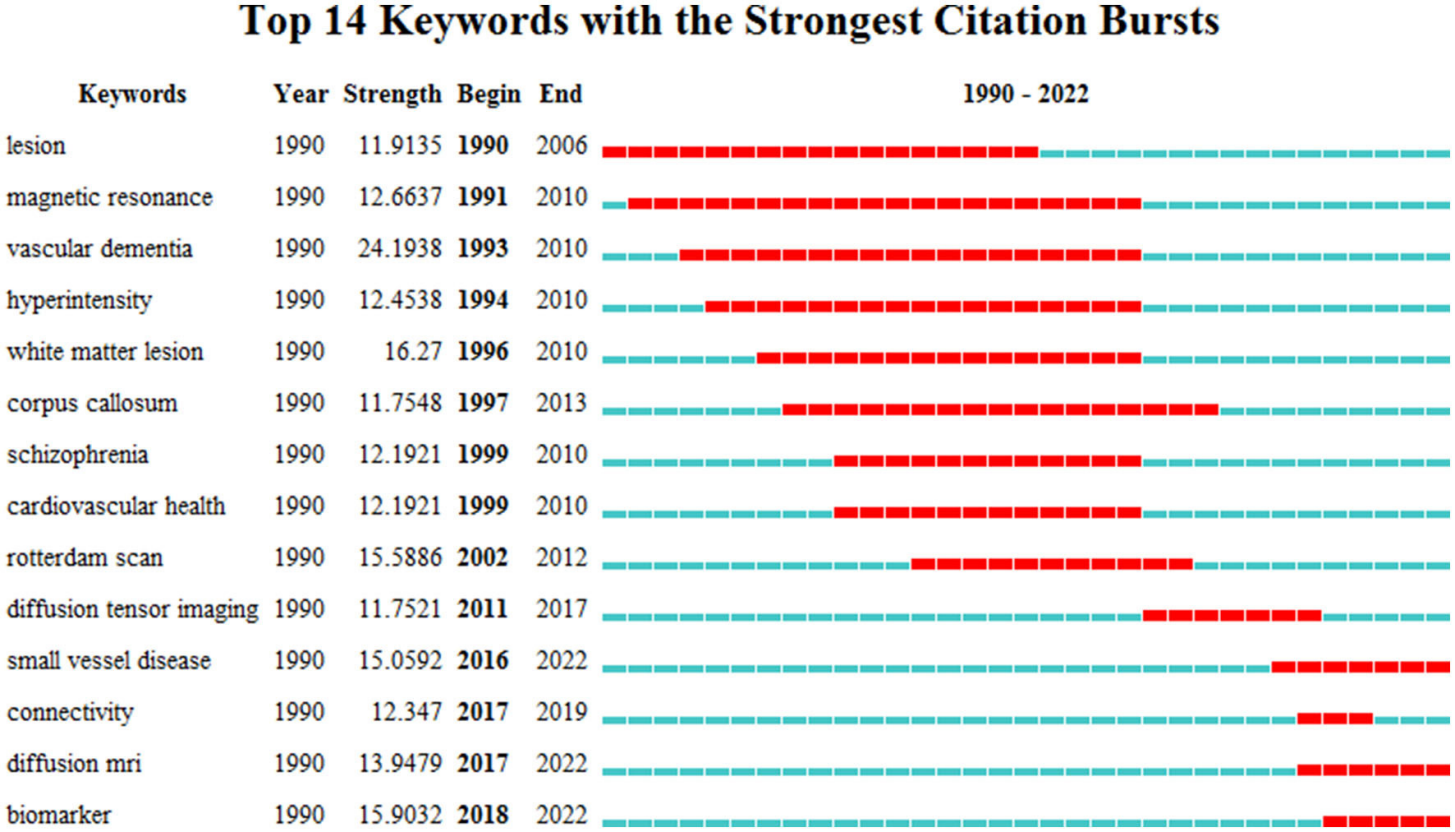
Top 14 keywords with the strongest citation bursts.

The results of the research trend were carried out through keyword citation bursts using the CiteSpace software, as shown in Figure 8. The main set in the process was the Minimum Duration (1 year) and γ (3.33). In the graph, Begin and End indicate the start and end times of the burst, respectively, and Red indicates the time span of the burst.

As can be seen from the diagram, “magnetic resonance” began in 1991 and lasted until 2010 with a burst intensity (12.6637), which was the longest burst duration, indicating the importance of MRI for research in this field. The highest burst intensity word was “vascular dementia” (24.1938) and then followed by “WM lesion” (16.2700). The burst time period of “small vessel disease,” “diffusion MRI,” and “biomarker” were 2016–2022, 2017–2022, and 2018–2022, respectively, and they all lasted to the present. This indicates that these are current and future research hotspots and trends.

## 4. Discussion

Data were obtained from MRI studies of WM in AD from 1990 to 2022 in the WOSCC database using the CiteSpace software. This study investigated the countries, institutions, authors, journals, keywords, and reference co-citations in this field and comprehensively explored the research status, hotspots, and trends in the present domain.

### 4.1. Research status of MRI studies of WM in AD

An increasing trend was observed in the number of publications in this field, with a fitted curve index of y = 4.1374e^0.1294x^. The average number of publications was 17.9. The United States was the most productive country, accounting for 44.52% of the total literature, followed by England, the Netherlands, China, and Italy, with relatively high-yielding outputs. There is a close cooperation network between countries. England, Canada, and France were the most influential countries.

Among the institutions, the University of California Davis was the most productive, accounting for 5.32% of the total number of studies. An institutional network is closely connected, forming a strong cooperative relationship. The University of California Davis, University of California Los Angeles, and Mayo Clinic had higher centrality, indicating a larger impact in this field. Research results from these institutions are of great significance for the development of this field.

From the authors' perspective, a good cooperative relationship has been established between authors in this field, and cooperation mainly revolves around high-yield authors. Among them, DeCarli et al. (2004) was the most productive author, accounting for approximately 3.91% of the total literature and is mainly involved in the development of mild cognitive impairment to dementia (DeCarli et al., 2004); the relationship between high-intensity WM volume and stroke, mild cognitive impairment, dementia, and mortality (Debette et al., 2010); the relationship between high-intensity WM around the ventricle, high-intensity WM in the deep and total WM load (DeCarli et al., 2005); and the diagnosis of AD using MRI (Cuenco et al., 2008).

Scheltens P was the author with the highest centrality, mainly involving the clinical application of structural MRI in AD (Frisoni et al., 2010); the relationship between high WM intensity in the elderly, cerebral amyloid angiopathy, and dementia (Tanskanen et al., 2013); the quantitative method of high WM intensity on MRI (Gao et al., 2011); and the WM change scale (Wahlund et al., 2001).

In terms of journals, Neurology accounted for 7.37% with an IF of 11.800 (2021), and it was the basis for the first 10 journals with the most influential factors. The journal with the highest number of citations was Neurology and the journal with the highest IF was Lancet Neurology (59.935). The journals with the highest centrality were Neurology (0.12) and Neurobiology of Aging (0.11). The study found that Neurology and Neuroimage were the top two journals in terms of the number of publications and total citations between 1990 and 2022, contributing to the development of the field.

### 4.2. Hotspots and research status on MRI of WM in AD

According to the keyword burst, the research fields of MRI on WM in AD were AD, MRI, dementia, brain, WM, mild cognitive impairment, WM hyperintensity, atrophy, WM lesions, and risk factors. In terms of centrality, age, MRI, lesion, healthy elderly subjects, and risk factors were all greater than 0.30, ranking among the top five most influential factors. The age of onset is a key factor in determining the obvious characteristics of patients with cognitive impairment, such as pathological burden and structural changes (Jang et al., 2016).

Based on clustering, the results were scientific and reliable, and were divided into nine types: #0 aging, #1 senile dementia, #2 magnetic resonance imaging, #3 mild cognitive impairment, #4 voxel-based morphometry, #5 leukoencephalopathy, #6 progression, #7 registration, and #8 cortex. WM lesions, mild cognitive impairment, high WM intensity, MRI findings, and risk factors were the main research focuses. These findings suggest that leukoencephalopathy is a potential risk factor for memory and cognitive impairment (No et al., 2019), whereas the synergistic effect between high WM intensity and hippocampal atrophy and the interaction between vascular and degenerative processes may be an important determinant of dementia (Wu et al., 2002), increased risk of neurological abnormalities associated with parietal region lacunae (parietal lobe space), and high-intensity WM (Camarda et al., 2015).

MRI is an important tool and method in the study of recognition, and it is of great significance in the diagnosis of AD with the aid of MRI. Structural MRI can explore the underlying pathophysiology through histopathology (Reijmer and Van Veluw, 2016), and functional magnetic resonance imaging (fMRI) can be used to detect brain function in patients (Yang et al., 2015), multimodal MRI is widely used to detect vascular cognitive impairment (Xia et al., 2022). Rs-fMRI can detect leukoaraiosis and a wide range of brain dysfunction (Cheng et al., 2017). In addition, other MRI techniques such as small vessel disease-related MRI, neuromelanin-sensitive MRI, diffusion-weighted imaging, cerebrovascular abnormality-related MRI, resting fMRI, and proton magnetic resonance spectroscopy can provide imaging features that can predict the degree of cognitive impairment in AD (Hou and Shang, 2022). Early studies have shown that mild cognitive impairment is a precursor to early clinical signs of AD, and changes in WM volume during this period may be of great value in clinical practice (Pergher et al., 2020). It has become the focus of epidemiology, neuroimaging, biomarkers, neuropathology, disease mechanisms, and clinical trials (Petersen et al., 2009).

From the perspective of reference co-citation, literature with high co-citation and centrality has been published in relatively high-influence international publications with high academic reference values. Among them, the research of highly co-cited literature mainly involves neuroimaging criteria of small vessel disease and its effect on aging and neurodegeneration (Wardlaw et al., 2013), the latest development of high-intensity WM, cognitive impairment, and dementia (Prins and Scheltens, 2015), the FSL (FMRIB software library) MRI brain imaging data comprehensive analysis tool library (Jenkinson et al., 2012), the composition of high-intensity WM (Wardlaw et al., 2015), and high-intensity WM as a core characteristic of MRI evidence of AD (Lee et al., 2016).

The high centrality studies were mainly related to the prevalence of WM lesions in the elderly (Leeuw et al., 2001), the potential risk factors and clinical manifestations of WM manifestations on brain MRI (Longstreth et al., 1996), the “Axial” and “Radial” diffusion rates of WM pathological regions (Wheeler-Kingshott and Cercignani, 2009), the changes of WM in mild cognitive impairment and AD (Medina et al., 2006), the age-related mental decline and high intensity of WM in healthy octogenarians (Garde et al., 2000).

In terms of keyword burst, the “magnetic resonance” burst lasted the longest, and the “vascular dementia” burst intensity was the largest at 24.1938. The burst time of “small vessel disease,” “diffusion MRI,” and “biomarker” were 2016–2022, 2017–2022, and 2018–2022, respectively, and they all lasted to the present. Best et al. identified increased perivascular space in the deep WM of the brain as a risk factor for intracranial hemorrhage in patients taking oral anticoagulants in a prospective initial cohort study (Best et al., 2020). Camarda's study assessed WM hyperintensities using the APOE genotype and normal cognition, mild cognitive impairment and AD brain imaging studies, and the visual scoring scale. The APOE ε3 allele may be associated with cerebrovascular diseases, especially in the frontal and parietal-occipital lobes (Camarda et al., 2022). In terms of diffusion MRI, Andersen's study showed that demyelination and axonal degeneration reduced fractional-order anisotropy in normal WM, which can be routinely imaged using diffusion tensor imaging (Andersen et al., 2020). In addition, researchers such as David S (David et al., 2022) and Chang YL (Chang et al., 2021) have used diffusion MRI. Neuroimaging biomarkers have been used in the clinical diagnosis, differential diagnosis, treatment, and prognosis of Parkinson's disease and AD (Li et al., 2022).

Gomar et al. (2011) reported that biomarkers are becoming increasingly important for understanding the neurodegeneration associated with AD (Gomar et al., 2011). Beaudin et al. (2022) suggested that small vessel disease and other vascular factors increase the risk of AD, and decreased cerebrovascular reactivity is a central feature of cerebral amyloid angiopathy, which can serve as another biomarker for disease severity and cognitive impairment (Beaudin et al., 2022). A prospective study of high-intensity MRI in WM and biomarkers such as neurofilament chain and glial fibrillary acidic protein revealed that serum GFAP is a promising fluid biomarker because it is associated not only with clinical severity but also with cognitive function (Huss et al., 2022). Ford et al. (2022) found that blood–brain barrier dysfunction is a hallmark of aging and aging-related diseases, including small vessel disease of the brain and AD. A novel biomarker of blood–brain barrier dysfunction is the blood–brain barrier water exchange rate [k(W)], as measured by diffusion-weighted arterial spin labeling (DW-ASL) MRI (Ford et al., 2022). In summary, the research field of MRI in WM of AD will focus on the association between small vessel disease and AD, and the clinical application and exploration of diffusion MRI. In particular, image marker research has a larger scope for further development.

### 4.3. Limitations

This study has several limitations. First, this study only obtained literature from the WOSCC database, and articles not covered by the WOSCC were not included. Second, all studies were published in English, and records in other languages were not included. Third, some original studies were manually reported; thus, some potential studies may have been lost. Fourth, the classification of clusters may be insufficiently precise because of the software.

## 5. Conclusion

This study investigated the current research status, hotspots, and frontier trends in MRI of WM in patients with AD from 1990 to 2022. The present research situation indicates that the research field of MRI in the WM of AD has great potential for development. Research hotspots include WM disorders, mild cognitive impairment, high-intensity WM, MRI, and risk factors mainly involved in neuroimaging, neuropathology, vascular pathology, and epidemiology. Research trends include the relationship between small vessel disease and AD, clinical applications and exploration of diffusion MRI, and research on related imaging markers.

## Author contributions

J-hY, Q-hZ, XY, and X-LL: concept and design. XY, PW, X-CS, and AL: data curation. XY and X-CS: formal analysis. X-LL, D-NC, L-WZ, and Q-hZ: funding acquisition. X-LL, L-WZ, and GY: investigation and project administration. XY, X-CS, and AL: methodology. J-hY, Q-hZ, XY, X-CS, Z-YW, and X-LL: resources. XY: software. Q-hZ, X-LL, and GY: supervision. All authors contributed to the article and approved the submitted version.
